# A ‘Standard of Care PLUS’ Model for Preterm Birth Prevention: Integrating Nutrient and Gene Variant Analysis with Targeted Interventions

**DOI:** 10.3390/jpm16030134

**Published:** 2026-02-28

**Authors:** Leslie P. Stone, Emily Stone Rydbom, P. Michael Stone, Daniel Kim

**Affiliations:** 1 Stone Medical PC, Ashland, OR 97520, USA; 2 GrowBaby Health, Ashland, OR 97520, USA; info@growbabyhealth.com; 3 Office of Personalized Health and Well-being at the AU/UGA Medical Partnership, University of Georgia, Athens, GA 30602, USA; mstone@ashlandmd.com; 4 Department of Research and Innovation, Ateneo School of Medicine and Public Health, Metro Manila 1604, Philippines; 5 Department of Mathematics, Southern Oregon University, Ashland, OR 97520, USA; kimd@sou.edu

**Keywords:** preterm birth, personalized nutrition, nutrigenomics, micronutrient deficiencies in pregnancy, epigenetics, gestational diabetes mellitus, hypertensive disorders of pregnancy, Standard of Care PLUS, maternal–neonatal outcomes

## Abstract

**Background/Objectives:** The rates of adverse maternal and neonatal outcomes—including preterm birth < 37 weeks’ gestation (PTB), hypertensive disorders of pregnancy (HDP), gestational diabetes mellitus (GDM), small for gestational age (SGA), and large for gestational age (LGA)—remain elevated in the United States. Preventive strategies beyond the current standard of care (SOC) may be needed, particularly in diverse and socioeconomically vulnerable populations. The study evaluated a targeted diet and lifestyle intervention incorporating selected nutrient and gene variant analysis with personalized trimester-based counseling and supplementation (Standard of Care Plus, PLUS). **Methods:** The prospective observational study compared outcomes among participants receiving PLUS in addition to SOC with regional SOC data. A Nevada PLUS cohort (*n* = 15), consisting of high-risk participants with 100% Medicaid coverage, received the intervention virtually. An Oregon PLUS cohort (*n* = 387), consisting of moderate-risk participants with approximately 50% Medicaid coverage, received PLUS through in-person group sessions. Outcomes were compared with regional SOC rates and between PLUS cohorts. Cochran–Mantel–Haenszel (CMH) analyses were performed to account for site-level differences in pooled analyses. Primary outcome was PTB < 37 weeks’ gestation; secondary outcomes included HDP, GDM, SGA, and LGA. **Results:** The Nevada PLUS application was associated with lower adverse outcome rates compared with regional SOC; however, statistical significance was not observed, likely reflecting limited sample size. The Oregon PLUS cohort experienced statistically significant association with reductions across all five outcomes (all *p* < 0.001) compared to regional SOC. No statistically significant differences were observed between the Nevada (virtual) and Oregon (in-person) PLUS cohorts. In pooled analyses (*n* = 402), significant reductions compared with SOC were observed for PTB (RR = 0.23), HDP (RR = 0.11), GDM (RR = 0.06), SGA (RR = 0.25), and LGA (RR = 0.35) (all *p* < 0.001). **Conclusions:** The implementation of selected nutrient and gene variant analysis combined with targeted nutritional and lifestyle interventions, delivered in collaboration with standard obstetric care, was associated with reduced adverse maternal and neonatal outcomes. Interpretation of virtual delivery remains limited by small sample size.

## 1. Introduction

The United States (US) faces a significant public health challenge with preterm birth (PTB) rates that are alarmingly high compared to other affluent nations. This disparity is particularly pronounced within socioeconomically disadvantaged communities and within racial minorities because of cost and geographic access to personalized health care, health literacy, non-primary language, provider shortages in underserved areas, and food deserts, among others [1]. As a result, a disproportionate burden of adverse outcomes falls in Medicaid-eligible populations specifically. In the US (2022), infant mortality rates due to prematurity/low birthweight account for 14.0% of all infant deaths [2].

The consequences of preterm birth extend far beyond the neonatal period and are associated with increased neurodevelopmental and cardiometabolic morbidity later in life [3,4]. Infants born small for gestational age (SGA), even at term, represent a distinct population at heightened risk for metabolic dysregulation and chronic disease development extending into adulthood [4,5]. These findings underscore the critical role of the intrauterine environment in shaping long-term health trajectories [3,4,5].

In the US, severe maternal morbidity (SMM) rose from 69.5 to 79.7 per 10,000 delivery hospitalizations between 2012 and 2019 [6]. During a similar time period, national obesity rates increased from 30.5% (1999–2000) to 44.9% (2017–2020), increasing risks for conditions like hypertension (HTN), diabetes (DM), and cardiovascular disease (CVD), as well as pregnancy-specific complications including hypertensive disorders (HDP), preeclampsia (PreE), preterm birth (PTB), gestational diabetes (GDM), and large for gestational age (LGA) infants [7]. Chronic HTN in pregnancy doubled between 2007 and 2021, with only 60% treated [8]. Uncontrolled HTN significantly raises risks for PreE, PTB, and SGA, which in turn increases future non-communicable disease (NCD) risk in offspring [8].

In 2022, PTB occurred in 10.4% of US births in 2022 and is associated with increased risk of maternal and offspring hypertension, cardiovascular disease, diabetes, osteoporosis, maternal complications of PTB, GDM, HDP, and neonatal SGA [9]. SGA occurred in 11.1% of the US population in 2021 and is associated with neurodevelopmental delay, infant and neonatal mortality, and chronic disease in the offspring [10]. LGA neonates comprised 11% of US neonates in 2018 [11] and are associated with an increased risk of insulin resistance, obesity, diabetes mellitus, early cardiovascular disease, and certain cancers [12]. The rising prevalence of non-communicable diseases among reproductive-age adults perpetuates a feed-forward cycle of chronic illness and adverse maternal and neonatal outcomes, disproportionately affecting populations experiencing racial, environmental, socioeconomic, and food access disparities [13].

### 1.1. Nutrition

While tackling the rising burden of NCDs is complex and multifactorial, predispositions appear modifiable in the perinatal period and provides an important opportunity for intervention. These include factors related to macro/micronutrient availability, gut microbiota, dietary composition (fatty acids, carbohydrates, protein), and toxic exposure [14]. Adequate nutrition during critical developmental windows significantly impacts fertility, pregnancy outcomes, and long-term maternal and offspring health [15,16]. However, prevalent Western dietary patterns are characterized by processed, calorie-dense foods, often low in essential micronutrients (e.g., iron, iodine, folate, B12, D, choline, and omega-3s) [15]. Access to nutrient-dense foods remains difficult for many women across socioeconomic levels, and, therefore, adequate and balanced nutrition during pregnancy cannot be assumed. As a result, interventions that address only a single nutrient are unlikely to be sufficient [15,17]. Standard obstetric care routinely screens for iron deficiency anemia but does not systematically evaluate other micronutrient deficiencies. When reproductive age norms are applied, preconception micronutrient deficiencies appear common and may increase the risk for adverse maternal and neonatal outcomes. In fact, 95% of pregnant women in the US fail to meet dietary recommendations for at least one nutrient through diet alone, with one in three remaining at risk even with supplements [15,18]. Iron [19,20,21], carnitine [22], zinc [23,24], and vitamin D [25,26,27,28] deficiencies are associated with many maternal and neonatal morbidities. Multiple micronutrient (MMN) supplementation reduces low birth weight by 12% (RR 0.88) and for SGA births by 8% (RR 0.92) and likely reduces preterm births by 5% (RR 0.95) [29]. Expert consensus supports MMN use from preconception through pregnancy, with particularly strong evidence for folic acid, iron, iodine, vitamin D, and docosahexaenoic acid (DHA) [30]. Importantly, giving DHA in pregnancy irrespective of maternal serum status decreases the risk of PTB [31]. Understanding how these prevalent nutritional deficiencies translate into significant health risks for both mother and child is paramount, and epigenetic processes provide a plausible framework for explaining individual variation in response to similar environmental and nutritional exposures.

### 1.2. Epigenetics

Pre- and post-transcriptional epigenetic processes may explain how gestational nutrition and environmental exposures impact immediate pregnancy outcomes and long-term mother/child disease risk. Optimizing both epigenetic and metabolic processes requires an interdependent network of micronutrients and vitamins, including iron, calcium, B vitamins, zinc, and magnesium, among others [32]. For example, methylation for these functions depends on dietary factors like betaine and choline, plus co-factors such as 5-methyltetrahydrofolate, vitamin B12, and other B vitamins such as riboflavin and niacin [32]. Schmidt et al. recognized that the application of intervention is also time-sensitive, finding that autism risk reduction in the first generation occurred if adequate methylation factors were provided 3 months before and one month after conception [33]. Additionally, risk appeared influenced by the number and origin (maternal or fetal) of select methylation-related gene variants involved [33]. The NiPPeR trial demonstrated that preconception and pregnancy micronutrient supplementation with folate, B12, B6, and other epigenetically active compounds can reduce preterm birth and early childhood obesity while inducing widespread epigenetic changes [34]. Vitamin D, omega-3 fatty acids (particularly DHA), and polyphenols regulate histone modifications and non-coding RNA expression [35].

To bridge the gap between burgeoning genomic data and tangible clinical benefits, the investigators undertook a systematic process of single-nucleotide polymorphism (SNP) selection based on the following criteria:

1. Strength of association with maternal and neonatal adverse outcomes under study;
2. Overlapping association with SNPs implicated in risk for chronic disease;
3. SNP frequency in the population;
4. Modifiability of the gene or gene product through nutrition, nutrient supplementation, and lifestyle modification.

To further personalize nutritional and lifestyle advice, 42 SNPs in 27 genes across 11 key biological processes were selected. Appendix A Table A8 summarizes the 11 biological processes represented in the panel, their constituent genes, and primary biological functions relevant to maternal and neonatal health. Although the selected panel reflects current evidence linking these biological processes to modifiable maternal and neonatal risk, nutrigenomic research in the perinatal period remains an evolving field. As the evidence base expands refinement of panel composition and corresponding recommendations are expected. These gene variants represent one component of a multifactorial risk landscape and do not independently predict outcomes; ongoing phenotypic biomarker assessment and clinical evaluation remain essential to individualized care.

**Figure 1:**
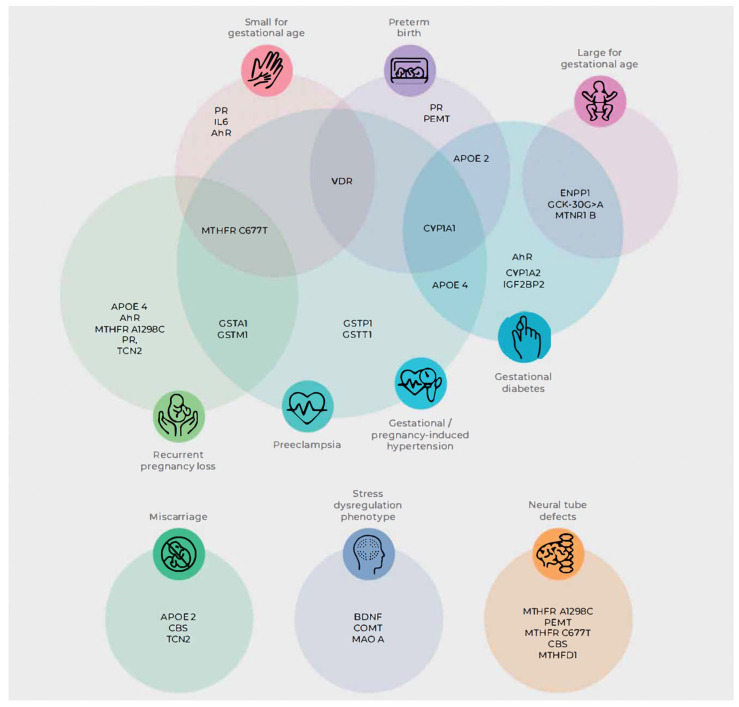
Gene single-nucleotide polymorphism with overlapping influences on pregnancy and fetal outcomes. Small for gestational age: Progesterone Receptor (PR), Interleukin-6 (IL6), Aryl Hydrocarbon Receptor (AhR), Vitamin D Receptor (VDR), Methylenetetrahydrofolate reductase C677T (MTHFR C677T); preterm birth: VDR, Apolipoprotein E-2 (APOE2), Cytochrome P450 Family 1 Subfamily A Member 1 (CYP1A1), PR, Phosphatidylethanolamine N-methyltransferase (PEMT); large for gestational age: Ectonucleotide pyrophosphatase/phosphodiesterase 1 (ENPP1), Glucokinase (GCK-30G>A), Melatonin Receptor 1B (MTNR1B); gestational diabetes mellitus: Cytochrome P450 Family 1 Subfamily A Member 2 (CYP1A2), Insulin Like Growth Factor 2 MRNA Binding Protein 2 (IGF2BP2), Apolipoprotein E-4 (APOE4), CYP1A1, APOE2; gestational/pregnancy-induced hypertension and preeclampsia: Glutathione S-Transferase Pi 1 (GSTP1), Glutathione S-transferase theta 1 (GSTT1), Glutathione S-Transferase Alpha 1 (GSTA1), Glutathione S-Transferase Mu 1 (GSTM1), MTHFR C677T, VDR, CYP1A1, APOE4; recurrent pregnancy loss: APOE4, AhR, Methylenetetrahydrofolate reductase A1298C (MTHFR A1298C), PR, Transcobalamin 2 (TCN2), GSTA1, GSTM1, MTHFR C677T; Miscarriage: APOE2, Cystathionine Beta-Synthase (CBS), TCN2; stress dysregulation phenotype: Brain Derived Neurotrophic Factor (BDNF), Catechol-O-Methyltransferase (COMT), Monoamine Oxidase A (MAO A); neural tube defects: MTHFR A1298C, PEMT, MTHFR C677T, CBS, Methylenetetrahydrofolate Dehydrogenase, Cyclohydrolase and Formyltetrahydrofolate Synthetase 1 (MTHFD1) [36].

Interventions, incorporated into time-sensitive plans, prioritized processes with multiple variants and high overlap with adverse maternal/neonatal outcomes and chronic disease risk. SNPs without diet or lifestyle remediation were excluded, and a polygenic risk score was not created (Figure 1).

### 1.3. Probiotic Mechanisms in Pregnancy

Maternal microbial ecosystems play critical roles in shaping reproductive physiology and pregnancy outcomes, modulating immune tolerance, nutrient metabolism, and susceptibility to complications, including preterm birth, hypertensive disorders, and gestational diabetes [37,38]. Pregnancy induces substantial alterations in the gut, vaginal, and oral microbiota driven by hormonal, immune, metabolic, and dietary factors [39]. The microbiome influences pregnancy through several interconnected mechanisms. Immune modulation occurs as the maternal microbiota regulates immune tolerance and inflammatory responses at the maternal–fetal interface, supporting stage-specific immunological balance across gestation, from the pro-inflammatory milieu favoring implantation and placentation to the anti-inflammatory environment required for fetal development [38,40]. Metabolic regulation is mediated through microbial production of key metabolites, including acetate, formate, and carnitine, which affect maternal and placental metabolism; notably, *Bifidobacterium* species promote placental morphogenesis, nutrient transporter capacity, and fetal growth [41]. Maternal gut dysbiosis disrupts these processes and is associated with increased systemic inflammation, impaired glucose metabolism, preeclampsia, preterm birth, and recurrent miscarriage [38,40]. The maternal microbiome further influences offspring health through fetal programming, impacting development of the fetal gut–brain axis and contributing to long-term health outcomes, including risk for allergic, neurodevelopmental, and metabolic disorders [42,43].

Probiotic supplementation, particularly multi-strain formulations containing *Lactobacillus* and *Bifidobacterium* species, has demonstrated anti-inflammatory, metabolic, and immunomodulatory actions with clinical relevance in pregnancy [44]. Umbrella reviews of randomized controlled trials report significant effects of probiotic interventions on maternal glycemic control, insulin metabolism, oxidative stress, and inflammatory marker reduction, with infant benefits including gut microbiome remodeling, reduced allergy and eczema risk, and decreased neonatal mortality [44].

### 1.4. A Personalized and Proactive Approach

It is in this context that a Standard of Care Plus (PLUS) model was conceived, augmenting current Standard of Care (SOC) by providing preventive collaborative nutritional counseling and lifestyle care in a highly personalized and time-cognizant manner. The PLUS model addresses specific gaps in the SOC model. PLUS identifies key micronutrient insufficiencies contributing to adverse outcomes by testing for zinc, carnitine, 25-hydroxy cholecalciferol (25(OH)D), and DHA. It recognizes individual metabolic vulnerabilities with select gene variant assessment. It postulates a role for probiotics in optimizing immune health and metabolic balance. These interrogations allow pro-active risk mitigation with personalized nutrition and lifestyle counseling led by a board-certified holistic nutritionist (BCHN).

The original 50% Medicaid, in-person group educational model in collaboration with SOC realized statistically significant reductions in aggregate occurrence of preeclampsia, GDM, SGA, and LGA compared to local private practice and community health clinic SOC [14]. The present study expands upon prior work by evaluating risk reduction for PTB, assessing a virtual delivery platform, including a larger and more diverse study population, and employing a prospective observational design. The objective of this study was to evaluate whether the PLUS intervention, delivered either virtually or in-person alongside standard obstetric care, was associated with reductions in PTB, HDP, GDM, SGA, and LGA compared with regional SOC populations. To our knowledge, this is the first study to deliver a personalized nutrigenomics-based prenatal intervention via a fully virtual platform to a Medicaid population.

## 2. Materials and Methods

### 2.1. Primary Application

Between 1 January 2011 and 31 December 2017, all pregnant women presenting for obstetric care at a private practice in Jackson County, Oregon, received the PLUS model of prenatal care at no cost to the patient. This model augmented standard obstetric care with in-person group sessions led by a BCHN. Sessions lasted up to 90 min per trimester and postpartum and included nutrition and lifestyle assessment, education, and development of individualized plans addressing diet, supplementation, and lifestyle factors. Plans were informed by health history, anthropometric measures, selected serum micronutrient levels, and selected gene variants. An additional 30 min per trimester and postpartum was allocated for individualized plan modification as needed. Postpartum support continued after delivery, and all births occurred in hospital settings. No exclusion criteria were applied.

### 2.2. Secondary Application

From 1 August 2022 to 1 July 2023, a prospective observational study was conducted at a private obstetric practice in Clark County, Nevada. Pregnant women at less than 20 weeks’ gestation with managed care organization (MCO) coverage received a cost-free virtual PLUS model. This model supplemented standard in-person obstetric care with six hours of individualized virtual assessment, education, and intervention delivered by a BCHN. The exclusion criteria included multiple gestations and fetal demise. All deliveries occurred in hospital settings and were completed by October 2023.

Educational materials were adapted to an eighth grade reading level and translated into Spanish. Nutrition and lifestyle interventions were delivered through HIPAA-compliant virtual communication and included an initial BCHN telephone consultation, three scheduled contacts per trimester and during the postpartum period, and unlimited participant-initiated text messaging. Clinic providers and staff received a 30-min virtual training session and an in-clinic logistical visit. Communication with obstetric providers regarding intervention modifications or nutrient-related diagnoses occurred via secure email. Data was stored in a separate, secure electronic health record system. Confidentiality protections include the de-identification of patient data, designated HIPAA-compliant data collection, storage and access by board certified investigators only and secured de-identified data handling.

Participant and publication consent in both primary and secondary application was procured in accordance with the Declaration of Helsinki 1964, and ethical review and approval was waived by the Institutional Review Board of Southern Oregon Internal Review Board. Informed consent was obtained to ensure patient understanding. Following consent, the participants underwent SOC evaluation by their obstetric provider, including height, weight, body mass index (BMI), and standard laboratory and imaging evaluation. In addition, each PLUS participant received the following assessments:

1. Nutritionist care consisting of 60 min per trimester delivered by phone or video conferencing;
2. Serum micronutrients zinc, carnitine, and 25(OH)D obtained at intake, 24–28-week gestation, and 6–8 weeks postpartum;
3. Dried blood spot DHA levels measured at intake, 24–28-week gestation, and breast milk DHA at 6–8 weeks postpartum;
4. Buccal swab collection for a 42-gene variant panel obtained at intake.

PLUS participants virtually received their individualized food and lifestyle plans based on obstetric provider-obtained anthropometric, laboratory and imaging assessments. Trimester-specific adaptations occurred based on subsequent PLUS testing and clinical response obtained with real-time chart review conducted by a board-certified physician investigator.

### 2.3. Interventions

The PLUS diet was based on a pregnancy-adapted Mediterranean Diet modified by a low glycemic index (low GI) with approximate macronutrient distribution of 40% carbohydrate/30% fat/30% protein ratio. Macronutrient requirements were calculated individually, and the low glycemic index, pregnancy-adapted Mediterranean diet food plan was adjusted each trimester according to nationally accepted pregnancy-specific Mifflin–St Jeor standards, with further refinement based on pre-pregnancy body mass index (BMI), gestational weight gain, activity level, singleton versus multiple gestation, and dietary preferences. Protein needs were calculated using 0.8–1.6 g/kg, then compared to basal metabolic rate and low GI macronutrient ratio to ensure repletion. Nutrition education was emphasized and outlined in Appendix A Table A1. Sleep quality, movement, exogenous stress and mood were assessed at intake, 24–28 weeks’ gestation, and 6–8 weeks postpartum. Common pregnancy-related concerns were addressed at each visit (Appendix A Table A2). Oregon PLUS patients received a multi-nutrient prenatal supplement pack (Appendix A Table A3) and probiotic (Appendix A Table A4). Nevada PLUS patients received a similar multi-nutrient pack (Appendix A Table A5) and separate, but similar probiotic (Appendix A Table A6). Customized vitamin D3 and iron supplementation was provided if identified needs could not be met with diet and lifestyle modification alone. Other micronutrient insufficiencies were managed with nutrient-rich food incorporation in the diet.

Compliance was evaluated by attendance tracking, customized food plan consumption and lifestyle adherence, a 5-food frequency questionnaire (Appendix A Table A11), and supplement consumption. Attendance tracking was recorded as percentage of the cohort that attended the intake, the second and third trimester, and the postpartum customized food and lifestyle plan creation and education. Compliance with the customized food plan and lifestyle recommendations was defined as following the plan five or more days per week relying on self-report and BCHN documentation. The 5-food frequency questionnaire was administered to the Nevada PLUS cohort at intake, second and third trimesters, and postpartum with a score of 4–5/5 recorded as high quality. Successful supplement compliance was defined as four or more days per week by self-report corroborated by refill frequency. Intervention comparisons are provided in Table 1.

The 42-SNP panel was utilized in the Nevada PLUS group to personalize diet and lifestyle recommendations versus a 2-SNP panel in the Oregon PLUS group. RS numbers, maternal and neonatal outcome associations, and prevalence in the Nevada PLUS population are included (Appendix A Table A9). The SNP datasets generated during the study are deposited in the National Center for Biotechnology Information dbSNP databank under BioProject accession number PRJNA1283159.

### 2.4. Primary and Secondary Outcomes

Two proportion Z tests were used to compare proportions between groups, and odds ratios (OR) and relative risks (RR) with corresponding confidence intervals were calculated. All personal data were de-identified prior to analysis. The primary outcome was the frequency of PTB, defined as delivery before 37 weeks’ gestation. Secondary outcomes included the frequency of HDP, GDM, SGA, and LGA, defined according to established diagnostic criteria (Appendix A Table A10). Outcomes were abstracted from electronic health and delivery records.

The frequency of each adverse outcome in the Oregon PLUS population was compared with the frequency observed in two comparator populations:

1. The Oregon State SOC population, as reported by the March of Dimes (2022), representing the regional standard of care comparator.
2. The Nevada PLUS population, representing the PLUS program comparator in a different geographic region and delivered through a different care model.

The frequency of each adverse outcome in the Nevada PLUS population was compared with frequencies observed in two comparator populations:

1. The Clark County, Nevada Medicaid SOC population, as reported by healthysouthernnevada.org (2021), representing the regional standard of care comparator.
2. The Oregon PLUS population, representing the program comparator in a different geographic region and delivered through a different care model.

To account for potential state level differences and heterogeneity between groups lacking individual level covariate data, including the SOC comparator populations for Nevada PLUS and Oregon PLUS, a secondary analysis was conducted using a state stratified Cochran–Mantel–Haenszel (CMH) method.

## 3. Results

### 3.1. Baseline and Comparative Characteristics

The Nevada PLUS study group and the Clark County, Nevada SOC population demonstrated similar distributions of age, geographic locality, gravidity, parity, race, BMI, and drug use, as reported by healthysouthernnevada.org (2021) [45]. However, the Nevada PLUS study group was 100% insured through Medicaid.

The Oregon PLUS study group shared several characteristics with the Nevada PLUS group, including gravidity, parity, and history of smoking, alcohol use, and drug use. However, the groups differed significantly in mean age (31.6 vs. 25.4 years, respectively) and racial composition (93.3 percent vs. 17 percent Caucasian, respectively) (Table 2). Medicaid coverage accounted for 100% of the Nevada PLUS group compared with 50% of the Oregon PLUS group.

The differences in BMI and excessive gestational weight gain between the two study groups warrant further consideration. Although the mean BMI at the first visit did not differ significantly between groups, the maximum BMI observed in the Nevada PLUS group was 35.1 kg/m^2^ compared with 54 kg/m^2^ in the Oregon PLUS group. A BMI greater than 30 kg/m^2^ was observed in 20% of the Nevada PLUS group and 11% of the Oregon PLUS group. Excessive weight gain, defined as greater than 40 pounds, occurred in 31% of the Nevada PLUS group compared with 25.4% of the Oregon PLUS group (Table 3).

### 3.2. Primary Application (Oregon PLUS vs. SOC)

The Oregon PLUS cohort was compared regionally to the statewide Oregon SOC population obtained from March of Dimes Report (2022). The SOC comparator data consist of aggregate counts rather than person-level observations, and individual-level covariates were not available. Both the primary and secondary analyses are presented as unadjusted effect estimates with corresponding 95% confidence intervals. Relative risk rates are descriptive, and not inferential.

The Oregon PLUS group experienced statistically significant associations with lower rates of all five adverse outcomes. Crude relative risks based on Oregon data alone are presented in Table 4.

**Figure 2:**
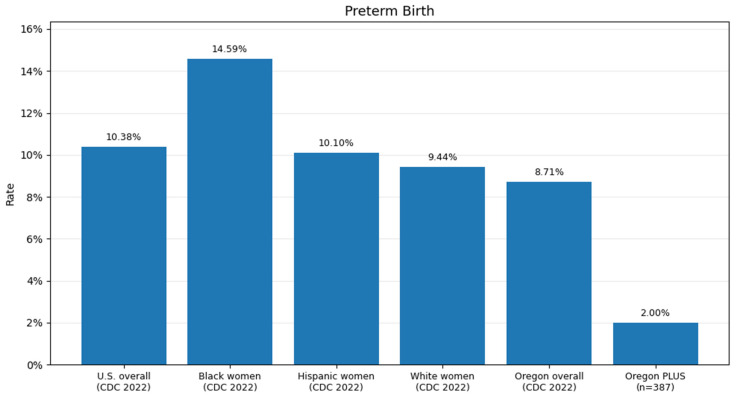
Comparative preterm birth rates: national, regional, and race/ethnicity-stratified data compared with Oregon PLUS.

Figure 2 compares regional and national preterm birth (PTB) rates, further stratified by Black and White race and Hispanic ethnicity, with those observed in the Oregon PLUS population. Preterm birth rates in the Oregon PLUS group were lower than corresponding regional and national rates across racial and ethnic strata. The SOC comparator data consist of aggregate counts rather than person-level observations, and individual-level covariates were not available.

### 3.3. Secondary and Pooled Analyses

#### 3.3.1. Nevada PLUS Descriptive Outcomes

In the Nevada PLUS cohort (*n* = 15), no events were observed for PTB, HDP, or small SGA, and one event was observed for GDM and LGA. Compared with contemporaneous Clark County, Nevada SOC rates (Southern Nevada Health District, 2021), outcome frequencies were descriptively lower in the Nevada PLUS group; however, statistical significance was not observed, likely reflecting the limited sample size. Direct comparison of Oregon PLUS and Nevada PLUS revealed no statistically significant differences in outcome rates, with effect estimates directionally consistent across sites.

#### 3.3.2. State-Stratified CMH Analysis (Pooled PLUS vs. SOC)

Given the absence of statistically significant differences in outcome rates between Oregon and Nevada PLUS cohorts, the two groups were pooled to increase statistical power for comparison with SOC. The pooled estimate represents the average effect of the PLUS intervention across two distinct populations: one socioeconomically diverse and fully Medicaid-covered (Nevada), and one more demographically homogeneous with mixed insurance coverage (Oregon).

State-stratified CMH analyses comparing pooled PLUS with SOC yielded common odds ratios below 1.0 for all five outcomes, indicating lower odds of adverse events in the PLUS group after adjustment for site. These associations remained statistically significant for all outcomes: preterm birth (OR 0.21, 95% CI 0.10–0.42; *p* = 1.47 × 10^−6^), hypertensive disorders of pregnancy (OR 0.21, 95% CI 0.07–0.60; *p* = 0.0015), gestational diabetes mellitus (OR 0.22, 95% CI 0.07–0.71; *p* = 0.0038), small for gestational age (OR 0.23, 95% CI 0.10–0.51; *p* = 8.54 × 10^−5^), and large for gestational age (OR 0.37, 95% CI 0.22–0.62; *p* = 7.69 × 10^−5^). Effect estimates were directionally consistent across Oregon and Nevada strata. Although the Nevada PLUS sample was small and included several zero-event cells, the consistent direction of effect across strata supports the appropriateness of pooling, while acknowledging the limited precision of Nevada-specific estimates.

#### 3.3.3. Numbers Needed to Treat

As an exploration, the numbers needed to treat (NNT) were calculated to provide clinical context for the magnitude of effect and to inform potential cost analyses. Across all five outcomes, NNT values ranged from 15 to 31, indicating that relatively few patients would need to receive PLUS to prevent one additional adverse event (Table 5). NNT values were calculated using pooled PLUS event rates relative to Oregon SOC reference rates, consistent with the primary comparator framework of this analysis.

### 3.4. Micronutrient and Macronutrient Analysis

Deficiency rates were compared between cohorts using two-proportion tests, with exact methods applied when small cell counts warranted. Outcomes included hemoglobin and hematocrit (surrogates for iron deficiency), serum zinc, carnitine, 25(OH)D, and dried blood spot DHA.

Regional and national pregnancy-specific comparators were limited, poorly standardized, or non-contemporaneous. Therefore, deficiency rates were compared with those reported for women of reproductive age (18–35 years) in the 2022 *Lancet* analysis of global, regional, and national burdens of common micronutrient deficiencies (1990–2019) [46] unless otherwise noted. Women of reproductive age were selected as the comparator group because they are age-matched and because physiologic changes in early pregnancy are less likely to substantially alter serum micronutrient concentrations during the first trimester.

In contrast to guideline-defined anemia thresholds, PLUS defined hemoglobin deficiency as Hb < 12 g/dL (12 g/dL is consistent with non-pregnancy reproductive-age females, CDC standard) to support earlier detection of iron-restricted physiology and early nutrition support rather than diagnosis of overt anemia. No meaningful difference in hemoglobin deficiency rate was found between the Nevada PLUS (16.7%) and Oregon PLUS (15.1%) populations (*p*-0.875). The national hemoglobin deficiency rate of 22% was not significantly different (*p*-0.074) [46].

Zinc deficiency was defined as less than 56 mcg/dL, and 50% of the Nevada PLUS cohort was zinc deficient while 37% of the Oregon PLUS population was deficient, representing no statistical difference between study groups (*p*-0.388). The national zinc deficiency rate of 14% was significantly less frequent (*p*-4.866 × 10^−8^) [46].

First trimester 25(OH)D insufficiency, defined as <30 ng/mL, occurred in 83% of the Nevada PLUS cohort, and 58% of the Oregon PLUS cohort. These occurrence rates are higher than the US-only weighted prevalence first trimester deficiency rate of 28% extracted from a global systematic review and meta-analysis [47]. We defined insufficiency as <30 ng/mL based on pregnancy-specific evidence demonstrating graded, quartile-dependent reductions in preterm birth, fetal growth restriction, and hypertensive disorders at higher maternal 25(OH)D concentrations, particularly when sufficiency is achieved in the first trimester [48,49,50,51]. Multiple prospective cohorts suggest early gestation represents a critical window, with risk attenuation extending beyond 20 ng/mL and lowest adverse outcome rates observed in higher 25(OH)D levels [48,49]. Given the disproportionate burden of vitamin D deficiency among populations with darker skin pigmentation and higher BMI, groups already at elevated obstetric risk, using a more conservative sufficiency threshold may better capture clinically meaningful vulnerability during pregnancy [52,53].

DHA deficiency rates were compared to reproductive-age females in an NHANES analysis during 2011–2012 [54]. The Nevada PLUS DHA deficiency (<5%) rate was 89% and was significantly higher than the national deficiency rate of 68% (*p*-0.029). DHA deficiency was defined as less than 5% content in red blood cells.

No meaningful regional, national or global comparator for carnitine was found [55], but both study populations were commonly deficient based on adult assay reference ranges, and not significantly different from each other. The free carnitine deficiency rate was 77.8% and 56.7%, Nevada PLUS and Oregon PLUS, respectively. The esterified carnitine deficiency rate was 55% and 53%, Nevada PLUS and Oregon PLUS, respectively. Free carnitine deficiency was defined as less than 20 µmol/L.

### 3.5. Compliance, Adherence, and Engagement

Eighty five percent of the Oregon PLUS in-person group implementation mode attended the intake customized food and lifestyle plan creation and education dropping to 70% attendance for second and third trimesters, and postpartum. Those attending the group visits remained compliant with customized food plans, lifestyle recommendations, and micronutrient supplementation by self-report and BCHN documentation.

Nevada PLUS participants were offered individual vs. group virtual nutritionist visits, with 100% opting for individual virtual visits. Intake attendance was comparable to the Oregon PLUS in-person group mode at 83%, decreasing to 47% by the third trimester and 45% during postpartum.

Nutrition/lifestyle adherence five or more days per week occurred in 78% of the Nevada PLUS cohort during the second trimester, diminishing to 53% by the third trimester. Food frequency scores maintained or improved to 4–5/5 from second to third trimester in 8 of 10 completed questionnaires. Postpartum questionnaire completion dropped to 4 out of 15 subjects, but all subjects maintained high scores (4–5/5). Qualitative data showed that the program aided food awareness and shopping choices persisting throughout pregnancy and postpartum. Eighty-to-ninety four percent of the cohort reported consuming their supplements four or more days per week throughout pregnancy and postpartum, corroborated by refill frequency.

Qualitative data showed that the program aided food awareness and shopping choices persisting throughout pregnancy and postpartum. Postpartum obstetric provider follow-up rates occurred in 5 out of 15 participants in the Nevada PLUS participants, while the Oregon PLUS follow-up exceeded 90%. These findings provide context for the clinical outcomes observed and inform the interpretation of the PLUS intervention within diverse population settings.

## 4. Discussion

The PLUS model integrated standard of care with targeted nutritional and lifestyle guidance beginning in the late first or early second trimester, guided by micronutrient assessment and genomic analysis and adjusted for physiologic changes during pregnancy. The PLUS model was associated with lower rates of PTB, HDP, GDM, SGA, and LGA compared with standard of care alone. The prespecified primary comparison (Oregon PLUS vs. Oregon SOC) demonstrated a substantially lower observed rate of adverse outcomes (RR 0.238). A state-stratified Cochran–Mantel–Haenszel (CMH) analysis combining Oregon and Nevada yielded a similar common effect estimate (OR 0.210), with no statistical evidence of heterogeneity across strata, supporting consistency across settings.

Secondary maternal and neonatal outcomes in pooled analyses were directionally consistent with benefit (RRs approximately 0.14–0.37). Although the Nevada PLUS cohort was small (*n* = 15) and individually underpowered, effect estimates were consistent in direction despite differences in age distribution, socioeconomic characteristics, and racial and ethnic composition.

Several issues in study design require refinement. Not all confounders are included in the analysis, such as self-selection bias completed years of education, primary language, socio-economic status beyond payer type, and environmental exposures. Causation cannot be established due to the observational design and lack of concurrent controls, which precludes adjustment for fundamentally different baseline risk profiles. It relies on some self-reported dietary and lifestyle compliance data. Individual strain-level colony forming units (CFU) dosing for the Oregon multi-strain probiotic blend was proprietary to the manufacturer and could not be independently verified, a common limitation in clinical research utilizing commercially formulated probiotics and may affect reproducibility. The current study design does not allow for the determination of which individual component(s) are responsible for the observed associations. It is possible that some or all of the observed association is attributable to increased clinical attention and engagement (Hawthorne effect) rather than specific nutritional or genomic components. Factorial trial design would be needed to parse individual component effects.

Despite the issues with study design the findings support the feasibility of the PLUS model across two distinct practice settings and justify further evaluation in a larger, adequately powered, multi-site prospective study incorporating individual-level covariates.

The virtual interface offers potential solutions for access disparities that limited participation in the 2011–2012 Oregon in-person model, including geographic, scheduling flexibility and other logistical concerns. After-hours communications were transitioned from the obstetrician to the nutritionist in the virtual model, potentially reducing physician time burden. Measures of intake attendance and second trimester adherence appeared comparable between in-person and virtual formats. The decline in third trimester and postpartum attendance in the virtual cohort may reflect reduced direct obstetric provider involvement or other unmeasured contextual factors. The virtual model offers advantages in scalability and earlier access to pregnant patients, but larger samples are needed to more robustly evaluate risk reduction and strategies for sustaining adherence. A human-centered, AI-driven digital health platform is under development to scale the PLUS model and to potentiate early access to health optimization for the reproductive age population.

Despite the demographic dissimilarities between the Oregon PLUS and Nevada PLUS groups, they shared remarkably common micronutrient insufficiencies for zinc, carnitine, and 25(OH)D. These micronutrient deficiency rates exceed national and global data suggesting that our current SOC screening practices are insufficient, leaving undetected nutritional vulnerabilities implicated in increased generational risk for non-communicable diseases. The limited availability of current validated pregnancy-specific biomarker reference ranges underscores the need for updated maternal standards. Reliance on dietary intake surveys alone may not adequately capture biologically meaningful nutrient status, particularly given documented changes in food composition and agricultural practices [56,57,58]. Furthermore, individual genetic variation may influence nutrient metabolism and bioavailability, suggesting that population-level recommendations may not fully address individual requirements.

The 42-SNP panel used in the Nevada PLUS cohort compared to the 2-SNP panel in the Oregon PLUS cohort may have contributed to similar outcome rates across demographically distinct groups, but larger datasets and multivariable modeling will be necessary to determine which components of the model are most influential. The investigators will extend this work through a prospective, longitudinal evaluation of a 500 dyad diverse mother–infant cohort with concurrent SOC control group, integrating early biomarker panels, nutrigenomic data, nutrition intervention exposure, and linked maternal–neonatal outcomes to assess durability of effect across pregnancy and the first five years of life. Primary pediatric outcomes will include developmental milestones, atopy, allergy, asthma, autism, and obesity evaluated at 1, 3 and 5 years. Furthermore, work is planned to expand understanding of nutrigenomic impact on generational health through interrogation of the imprintome [59,60,61].

While continued mechanistic investigations will deepen understanding of biological durability and intergenerational effects, parallel evaluation of clinical outcomes and health system impact is essential to contextualize the real-world value of the PLUS model. Comparing improved outcomes in PTB, SGA, LGA, GDM, and HDP under the PLUS model to US average rates and associated costs, investigators project potential annual gross savings [62,63,64,65,66,67]. Based on 3.6 million births per year [68], national adoption of the PLUS model is estimated to save the US healthcare system between USD 4.435 billion (NNT model) and USD 43.42 billion (percent reduction model) annually. Although this study is limited to short-term outcomes, longitudinal maternal and neonatal health assessment would provide a more comprehensive evaluation of the PLUS model’s generational impact on health and disease.

## 5. Conclusions

These results suggest that late first/early second trimester application of targeted nutrition, and lifestyle guidance based on select micro/macronutrient and genomic analysis, further adjusted for changing physiologic pregnancy need may be associated with reductions in the incidence of PTB, HDP, GDM, SGA, and LGA neonates when applied in collaboration with standard obstetric care. An association of lower rates of adverse maternal and neonatal outcomes was consistently observed over seven years in the Oregon PLUS group. The Nevada PLUS group association with lower rates of adverse outcomes was statistically indistinct from the Oregon PLUS group, suggesting that with rigorous and adequately powered study design, and results that confirm these observational findings, risk reduction of all adverse outcomes may be achievable in diverse and socio-economically challenged populations. In the meantime, several clinical actions are suggested: 1. routine screening for zinc, vitamin D, DHA, and carnitine alongside prenatal labs; 2. integrating preventative nutrition counseling as a standard component of obstetric care; and 3. considering Medicaid coverage of expanded micronutrient panels and nutrition counseling.

## Figures and Tables

**Table 1:** Intervention comparisons.

Component	SOC	PLUS Oregon	PLUS Nevada
Routine Nutrition Professional	None	In-person group: 90 min per trimester plus additional 30 min as needed	Virtual sessions 60 min per trimester plus additional 30 min as needed
Meal Plan	None	Initiated at any trimester upon first intake, with individual needs adjusted throughout pregnancy.	Initiated up to 22 weeks gestational age consistent with eligibility criteria, with individual needs adjusted throughout pregnancy
Prenatal Vitamins	Prenatal with iron and folic acid	Prenatal nutrient packet (Appendix A Table A3) taken daily from the 1st trimester (or earliest possible) through postpartum	Prenatal nutrient packet (Appendix A Table A5) taken daily from first trimester or earliest possible through postpartum
Probiotic	None	Probiotic (Appendix A Table A4) taken daily from the 1st trimester (or earliest possible) through postpartum	Probiotic (Appendix A Table A6) taken daily from first trimester or earliest possible through postpartum
Standard Labs	1st, 2nd, 3rd Trimesters (Appendix A Table A7)	SOC Labs	SOC Labs
Micronutrient Labs	None	Serum zinc, carnitine (free, total, acyl), 25(OH)D drawn at intake, 24–28-week gestation, and 6–8 weeks postpartum.	Serum zinc, carnitine, and 25(OH)D were tested at intake, 24–28 weeks, and 6–8 weeks postpartum; DHA was measured from dried blood spots (intake) and breast milk (6–8 weeks postpartum)
Nutrigenomics	None	MTHFR C677T and MTHFR A1298C	42 SNPs across 27 genes (Appendix A Table A9)

**Table 2:** Comparative characteristics of Nevada PLUS and Oregon PLUS.

Characteristic	Nevada PLUS (*n* = 15)	Oregon PLUS (*n* = 387)	*p*-Value
Age (years)	25.4 (SD 5.187)	31.6 (SD 5.378)	0.00038
Advanced Maternal Age (%)	0	29	
Teen (%)	20	0.74	
Gravidity	2.4 (SD 1.665)	2.97 (SD 1.76)	0.214
Parity	1 (SD 1.264)	1.13 (SD 1.17)	0.7
Race (%)			
Black	33	<1	
Hispanic	28	5	
Asian	11	<1	
Native American	0	<1	
Not Specified	11	0	
Caucasian	17	93.3	
Smoking, alcohol, drug history (%)	22	21.7	
Payer Source	100% Medicaid	50% Medicaid	

Continuous variables were compared using Welch’s *t*-test. Categorical variables are presented descriptively as percentages; no hypothesis testing was performed for categorical variables.

**Table 3:** Body mass index (BMI) and excessive gestational weight gain (EGWG) descriptive statistics in Nevada (SOC Plus) and Oregon (SOC Plus).

Characteristic	Nevada PLUS	Oregon PLUS
BMI at First Visit		
Mean	25.37 (4.716)	24.8 (SD 5.3)
Minimum	17.72	17
Maximum	35.1	54
BMI Ranges:		
25–30	27%	33%
>30	20%	11%
EGWG > 40 lbs	31%	25.4%

Notes: Continuous variables were compared using Welch’s *t* test. Categorical variables are presented descriptively as percentages; no hypothesis testing was performed for categorical variables.

**Table 4:** Observed rate ratio of adverse outcomes: Oregon PLUS compared to Oregon SOC.

Outcome	Oregon PLUS (*n* = 387)	Oregon SOC (*n* = 553)	*p*-Value	Relative Risk (PLUS vs. SOC)	Inverse RR (SOC vs. PLUS)
Preterm Birth	2.0% (8/402)	8.7% ^1^	<0.0001	0.238	4.20
Hypertensive Disorders	1.0% (4/402)	4.5% ^2^	0.0023	0.229	4.37
Gestational DM	0.5% (2/402)	3.7% ^2^	0.0006	0.071	14.00
Small for Gestational Age	1.5% (6/402)	6.1% ^1^	0.0006	0.252	3.97
Large for Gestational Age	3.5% (14/402)	9.4% ^1^	0.0003	0.357	2.80

^1^ March of Dimes, Oregon, 2022. ^2^ GAHMJ, November 2014, vol. 3;6. *n* = 553, combined community clinic and private practice deliveries 2011–2012 in the same hospital as Oregon PLUS.

**Table 5:** Numbers needed to treat (NNT) for pooled PLUS (Oregon and Nevada) compared with Oregon SOC.

Outcome	PLUS (*n* = 402)	SOC	NNT
Preterm birth	1.99%	8.7% ^1^	15
Hypertensive disorders of pregnancy	1.00%	4.5% ^2^	29
Gestational diabetes mellitus	0.50%	3.7% ^2^	31
Small for gestational age	1.49%	6.1% ^1^	22
Large for gestational age	3.48%	9.4% ^1^	17

Sources: ^1^ March of Dimes. Oregon Report Card, 2022. ^2^ Global Advances in Health and Medicine (GAHMJ). November 2014; 3(6): *n* = 553, combined community clinic and private practice deliveries (2011–2012) at the same hospital as Oregon PLUS.

## Data Availability

The SNP datasets generated during the study are deposited in the National Center for Biotechnology Information dbSNP databank under BioProject accession number PRJNA1283159. All other data are held in a dedicated secure device and can be made available upon request.

## Abbreviations

The following abbreviations are used in this manuscript:

APC	Article Processing Charge
BCHN	Board-Certified Holistic Nutritionist
BMI	Body mass index
CDC	Centers for Disease Control and Prevention
CFU	Colony forming units
CMH	Cochran–Mantel–Haenszel
CNP	Certified nutrition professional
DHA	Docosahexaenoic acid
DM	Diabetes mellitus
EGWG	Excessive gestational weight gain
GDM	Gestational diabetes mellitus
HDP	Hypertensive disorders of pregnancy
HTN	Hypertension
IRB	Institutional Review Board
LGA	Large for gestational age
MDHc	Master of Digital Health Candidate
NCD	Non-communicable disease
NNT	Numbers needed to treat
OGTT	Oral glucose tolerance test
PLUS	Standard of Care PLUS
PTB	Preterm birth (<37 weeks’ gestation)
SGA	Small for gestational age
SMM	Severe maternal morbidity
SOC	Standard of care
SNP	Single-nucleotide polymorphism
US	United States
25(OH)D	25-Hydroxyvitamin cholecalciferol

## Appendix A

**Table A1:** PLUS Nutrients by Trimester for Maternal & Fetal Health.

Trimester	Fetal Development Emphasis	Maternal Emphasis
**1**	**Protein:** Methionine, cysteine **Fats:** DHA **Minerals:** Iron, Magnesium **Vitamins:** A, D, B Complex, Choline	**Protein:** Carnitine **Carbohydrates:** Soluble & insoluble fiber **Minerals:** Iodine, Iron, Magnesium, Selenium **Vitamins:** A, C, D, B6 **Phytonutrients:** Rainbow of foods
**2**	**Fat:** DHA **Minerals:** Boron, Calcium, Iron, Magnesium, Molybdenum, Phosphorous **Vitamins:** A, D, E, B Complex **Phytonutrients:** Rainbow of foods carotenoids	**Protein:** Carnitine, glycine, lysine **Carbohydrates:** Soluble & insoluble fiber **Minerals:** Iron, Magnesium, Zinc **Vitamins:** A, C, D, B Complex **Phytonutrients:** Rainbow of foods, carotenoids
**3**	**Fats:** EPA & DHA **Minerals:** Copper, Iodine, Iron, Magnesium, Selenium, Zinc **Vitamins:** Choline, A, D, E, B Complex **Phytonutrients:** Rainbow, Carotenoids	**Protein:** Carnitine, glycine, lysine **Carbohydrates:** Soluble & insoluble fiber **Fats:** EPA, DHA, SPMs **Minerals:** Copper, Iodine, Iron, Magnesium, Selenium **Vitamins:** Choline, A, D, E, B Complex **Phytonutrients:** Rainbow of foods, carotenoids **Probiotics:** *L. Gasseri*, *L. Fermentum*, *L. Reuteri*, *L. Salivarius*, *L. Helveticus*, *B. Longum*
**4**	**Fat:** DHA **Minerals:** Calcium, Copper, Iron, Magnesium, Selenium, Zinc **Vitamins:** Choline, A, D, E, B Complex Phytonutrients: Rainbow of foods, Lutein, Zeaxanthin **Probiotics:** *L. Reuteri*, *L. Rhamnosus*, *B. lactis*, *B. Longum*	**Fat:** DHA & SPMs **Minerals:** Iron, Magnesium **Vitamins:** Choline, Inositol, D, B Complex **Phytonutrients:** Rainbow of foods, carotenoids **Probiotics:** *L. Gasseri*, *L. Fermentum*, *L. Reuteri*, *L. Salivarius*, *L. Helveticus*, *B. Longum*

**Table A2:** PLUS: Select common pregnancy/postpartum concerns with select nutrition and supplement recommendations.

Trimester	Select Symptoms of Pregnancy/Postpartum	Nutrition and Lifestyle Recommendations	Select Supplement Recommendations
1	Nausea	Eating frequency, small meals, protein emphasis, limit high histamine foods	Ginger root, B6
	Fatigue	Protein emphasis, iron metabolism support, and sleep support	Carnitine, iron, vitamin C, vitamin A, B vitamins
2	Constipation	Adequate fiber, hydrating foods/fluid/kiwi fruit, phytonutrient intake, pre/probiotic rich foods	Magnesium, fiber
	Restless Legs	Magnesium-rich foods, iron rich foods, electrolyte replenishment, coconut water	Magnesium, iron
	Heartburn	Limit antagonists: spicy, processed, gluten, dairy, sugar, caffeine and focus on alkaline rich foods/fluid, positional recommendations, almonds, apple cider vinegar, increase foods high in digestive enzymes (pineapple, papaya) as tolerated	Digestive enzymes
3	Insomnia	Limit stimulants sugar/caffeine, meal timing, support healthy melatonin secretion habits during daytime	Magnesium
4	Mastitis	Hydration, lymphatic support, anti-inflammatory foods	Lecithin, SPMs, *L. Gasseri*, *L. Fermentum*, *L. Salivarius*

**Table A3:** PLUS base nutrients (Oregon cohort).

Nutrient	Amount per Serving	% Daily Value for Pregnant and Lactating Women
Calories	10	—
Total Fat	1 g	2%
Vitamin A (Natural mixed carotenoids)	5000 IU	100%
Vitamin C (Ascorbic acid)	250 mg	417%
Vitamin D3 (Cholecalciferol)	2000 IU	500%
Vitamin E (d-Alpha tocopheryl acid succinate; d-Alpha and mixed tocopherols)	110 IU	367%
Thiamine/Vitamin B1 (Thiamine mono nitrate)	5 mg	333%
Riboflavin/Vitamin B2 (Riboflavin-5-phosphate)	5 mg	294%
Niacin/Vitamin B3 (Niacinamide)	25 mg	125%
Vitamin B6 (Pyridoxine HCl and Pyridoxal-5-phosphate)	15 mg	750%
Folate (L-5-Methyltetrahydrofolate, glucosamine salt)	1000 mcg	250%
Vitamin B12 (Methylcobalamin)	800 mcg	13.333%
Biotin (Biotin)	100 mcg	33%
Pantothenic Acid/Vitamin B5 (d-Calcium pantothenate)	25 mg	250%
Iron (Iron bis-glycinate)	25 mg	139%
Iodine (Potassium iodide)	150 mcg	100%
Zinc (Zinc citrate)	25 mg	167%
Selenium (L-Selenomethionine)	100 mcg	143%
Copper (Copper gluconate)	1.5 mg	75%
Manganese (Manganese gluconate)	5 mg	250%
Chromium (Chromium niacinate)	200 mcg	167%
Molybdenum (Molybdenum glycinate)	25 mcg	33%
Omega-3 Fatty Acids (Fish oil)	700 mg	**
EPA (Eicosapentaenoic acid)	500 mg	**
DHA (Docosahexaenoic acid)	200 mg	**
L-Carnitine (L-Carnitine tartrate)	1000 mg	**
Probiotic Blend (Freeze-dried cultures)	60 billion CFU	**

** daily value not established.

**Table A4:** PLUS base probiotic (Oregon cohort).

Probiotic Strain (Form)	Amount per Serving	% Daily Value
*Lactobacillus acidophilus* (La-14)	†	**
*Bifidobacterium lactis* (Bl-04)	†	**
*Lactobacillus plantarum* (Lp-115)	†	**
*Lactobacillus salivarius* (Ls-33)	†	**
*Streptococcus thermophilus* (St-21)	†	**

† Total blend provides 60 billion CFU. ** daily value not established.

Probiotic supplementation in the Oregon PLUS cohort consisted of a multi-strain blend providing 60 billion CFU total per serving as freeze-dried cultures in single oral capsule form, as above.

**Table A5:** PLUS base multi-nutrient pack (Nevada cohort).

Nutrient	Amount per Serving	% Daily Value for Pregnant and Lactating Women
Vitamin A (from mixed carotenoids and retinyl palmitate)	1650 mcg	127%
Vitamin C (as ascorbic acid and niacinamide ascorbate)	250 mg	208%
Vitamin D (as cholecalciferol)	25 mcg (1000 IU)	167%
Vitamin E (from mixed tocopherols and d-alpha tocopherol succinate)	90 mcg	528%
Vitamin K (as phytonadione (ISP)	90 mcg	100%
Thiamin (as thiamin mononitrate)	3.4 mg	243%
Riboflavin	3.4 mg	213%
Niacin (as niacinamide ascorbate)	35 mg	194%
Vitamin B6 (as pyridoxine HCl and pyridoxal-5-phosphate)	20 mg	1000%
Folate (as calcium L-5-methyltetrahydrofolate)	1700 mcg DFE	283%
Vitamin B12 (as methyl cobalamin)	800 mcg	28,571%
Biotin	300 mcg	857%
Pantothenic Acid (as calcium D-pantothenate)	20 mg	286%
Choline (as choline bitartrate)	450 mg	82%
Iron (as ferrous bis-glycinate)	30 mg	111%
Iodine (as potassium iodide)	176 mcg	61%
Zinc (as zinc citrate)	25 mg	192%
Selenium (as seleno-methionine)	200 mcg	286%
Copper (as copper citrate)	2 mg	154%
Manganese (as manganese citrate)	2 mg	77%
Chromium (as chromium citrate)	200 mcg	444%
Molybdenum (as molybdenum aspartate complex)	25 mcg	50%
DHA (Docosahexaenoic acid)	1 g	**
EPA (Eicosapentaenoic acid)	670 mg	**
L-Carnitine (as L-carnitine L-tartrate)	500 mg	**
Lutein	6 mg	**
Zeaxanthin	2 mg	**

** daily value not established.

**Table A6:** PLUS probiotic supplement (Nevada cohort).

Ingredient	Amount per Capsule	Daily Value
Serving Size: 1 capsule		
*Lactobacillus rhamnosus GR-1*	1 billion CFU ^1^	**
*Lactobacillus reuteri RC-14*	1 billion CFU ^1^	**

^1^ Colony-forming units/** daily value not established.

The Nevada PLUS cohort received a separate probiotic providing 2 billion CFU total per capsule, as above. One capsule was consumed orally each day.

**Table A7:** SOC Labs.

First trimester: Initial comprehensive blood work (blood type, Rh factor, CBC, infectious disease screening like HIV, Hepatitis B, Syphilis, Rubella immunity, urine culture). Third trimester: Glucose screening for gestational diabetes (typically 24–28 weeks), repeat CBC if indicated. Third trimester: Group B Strep (GBS) screening (typically 36–38 weeks), repeat labs if indicated (e.g., for preeclampsia concerns).

**Table A8:** Selected gene variants across 11 biological processes in the PLUS 42-SNPs across 27 genes.

Biological Process †	Genes	Primary Biological Function
**Lipid metabolism**	APOE	Lipoprotein catabolism and receptor binding; isoforms alter lipid transport, placental lipid delivery, and oxidative stress response [69,70,71,72,73,74].
**Inflammation**	IL-6	Encodes interleukin-6, a pro-inflammatory cytokine regulating CRP expression; mediates implantation, placental development, and fetal growth; dysregulation is associated with pathological pregnancy [75,76,77].
**Detoxification—Phase I**	AhR, CYP1A1, CYP1A2	Xenobiotic sensing (AhR) and cytochrome P450–mediated Phase I biotransformation; activates environmental procarcinogens and metabolizes estrogens and caffeine [72,78,79,80].
**Detoxification—Phase II**	GSTA1, GSTP1, GSTM1, GSTT1	Glutathione S-transferase–mediated conjugation and neutralization of reactive intermediates and products of oxidative stress [81,82,83].
**Methylation**	CBS, CHDH, COMT, MTHFR, MTHFD1, MTRR, PEMT, TCN2	One-carbon metabolism, homocysteine regulation, DNA methylation, neurotransmitter catabolism, choline/phosphatidylcholine synthesis, and vitamin B12 transport [32,33,84,85,86,87,88].
**Monoamine oxidase metabolism**	MAO-A	Degradation of monoamine neurotransmitters (dopamine, serotonin, norepinephrine); modulates maternal stress regulation; placental MAO expression is implicated in oxidative stress pathways in preeclampsia [89,90].
**Neurotrophic pathway**	BDNF	Brain-derived neurotrophic factor supports neuronal survival, differentiation, and synaptic plasticity; modulates maternal mood, stress response, and fetal neurodevelopment; maternal serum levels vary across pregnancy [91,92,93].
**Progesterone metabolism**	PROGINS	Encodes the progesterone receptor; PROGINS variants diminish progesterone response, affecting uterine function and implantation; effects may be modified by ethnic background, nutritional status, and gene–environment interactions [94,95,96].
**Melatonin receptor metabolism**	MTNR1B, MIR194-2 near MTNR1B	Melatonin receptor 1B mediates circadian and reproductive actions of melatonin; variants influence glucose regulation and insulin secretion in accordance with diurnal cycles [97].
**Insulin sensitivity, secretion and metabolism**	ENPP1, GCK, IGF2BP2, SLC30A8	Glucose phosphorylation (GCK), insulin receptor signaling (ENPP1), IGF2 mRNA regulation (IGF2BP2), and zinc-mediated insulin secretion (SLC30A8) [72,98,99].
**Vitamin D requirements**	VDR	Vitamin D receptor regulates calcium homeostasis, immune modulation, cell proliferation, and placental function; VDR polymorphisms modify maternal vitamin D concentration and neonatal outcomes [25,26,27,28,100,101,102].

**†** Select genotypes within these biological processes may confer protective rather than risk-increasing effects on associated outcomes; directionality is genotype-dependent. **Note**: In the Nevada PLUS cohort, investigators tested 42 SNPs in 27 genes. The Oregon PLUS cohort tested 2 SNPs (MTHFR C677T and A1298C).

**Table A9:** RS numbers, maternal and neonatal outcome associations, and prevalence in the Nevada PLUS population.

Gene	rs Number	SNP	Outcome Association					Prevalence
			PTB	HDP	GDM	SGA	LGA	(%)
AhR	2,066,853	Arg554Lys (G/A)	X	X		X		61
APOE	7412	E2/E2	X		X	X		0
APOE	429,358	E2/E3	X		X			30
		E2/E4		X	X			11
		E3/E3						39
		E3/E4		X	X			6
		E4/E4		X	X			11
BDNF	6265	Val66Met (C/T)						22
CBS	4,920,037	G>A						11
CBS	234,715	G>T						11
CHDH	12,676	Leu78Arg(A/C)						44
COMT	4818	C>G						100
COMT	6269	A>G						94
COMT	4633	C>T						72
CYP1A1	4,646,903	T>C	X	X	X			67
CYP1A1	1,048,943	A>G	X	X	X			33
ENPP1	997,509	C>T			X		X	6
GCK	1,799,884	-30G>A			X		X	50
GSTA1	3,957,357	C>T		X				50
GSTP1	1695	Ile105Val (A>G)		X				50
GSTM1	1,065,411	Insertion/Deletion		X				17 (deletion)
GSTT1	1,130,990	Insertion/Deletion		X				6 (deletion)
IGF2BP2	4,402,960	G>T				X		50
IL6	1,548,216	G>C				X		33
IL6	2,069,843	G>A				X		33
IL6	6,963,444	A>G				X		22
IL6	7,784,987	G>A				X		22
IL6	3,087,221	C>T				X		17
MAO-A	1,137,070	C>T						78
MAO-A	6323	G>T						37
MTHFR	1,801,131	1298A>C		X		X		44
MTHFR	1,801,133	677C>T		X		X		39
MTHFD1	2,236,225	Arg653zGln (G/A)				X		61
MTNR1B	10,830,963	C>G			X		X	44
MIR194-2 near MTNR1B	1,387,153	C>T			X		X	50
MTRR	1,801,394	66 A>G	X	X				67
PEMT	1,108,579	C>T	X					56
PR	11,224,592	C>T	X					55 (not protective)
PR	10,895,068	331G>A	X					6 (not protective)
TCN2	1,801,198	G>C						61
VDR	2,228,570	Fokl T>C	X	X	X	X		44
VDR	11,568,820	C>T	X	X		X		56
VDR	2,853,559	A>G	X	X		X		89
SLC30A8	11,558,471	G>A			X			94

Aryl Hydrocarbon Receptor (AhR); Apolipoprotein (APOE); Brain Derived Neurotrophic Factor (BDNF); Cystathionine Beta-Synthase (CBS); Choline Dehydrogenase (CHDH); Catechol-O-Methyltransferase (COMT); Cytochrome P450 Family 1 Subfamily A Member 1 (CYP1A1); Cytochrome P450 Family 1 Subfamily A Member 2 (CYP1A2); Ectonucelotide pyrophosphatase/phosphodiesterase 1 (ENPP1); Glucokinase (GCK); Glutathione S-Transferase Alpha 1 (GSTA1); Glutathione S-Transferase Mu 1 (GSTM1); Glutathione S-Transferase Pi 1 (GSTP1); Glutathione S-transferase theta 1 (GSTT1); Insulin Like Growth Factor 2 MRNA Binding Protein 2 (IGF2BP2); Interleukin-6 (IL6); Melatonin Receptor 1B (MTNR1B); Methylenetetrahydrofolate reductase (MTHFR); Methylenetetrahydrofolate Dehydrogenase Cyclohydrolase and Formyltetrahydrofolate Synthetase 1 (MTHFD1); Monoamine Oxidase A (MAO A); MicroRNA 194-2 (MIR194-2); Methionine Synthase Reductase (MTRR); Phosphatidylethanolamine N-methyltransferase (PEMT); Progesterone Receptor (PR); Transcobalamin 2 (TCN2); Vitamin D Receptor (VDR); Solute Carrier Family 30 Member 8 (SLC30A8).

**Table A10:** Defined diagnostic criteria.

Hypertensive disorders of pregnancy diagnosis and management based on American Academy of Obstetricians and Gynecologists (ACOG) Practice Bulletin No. 222. Obstet Gynecol June 2020; Vol 135(6): e-237-e260. Gestational diabetes mellitus diagnosis and management based on ACOG Practice Bulletin No. 190. Obstet Gynecol Feb 2018; Vol 131 (2): e49-e64. Small for gestational age condition defined by American Academy of Pediatrics gestational age condition defined by AAP standard as growth greater than or equal to the 90th percentile based on gestational age at delivery.

**Table A11:** Food frequency.

The 5-food frequency questionnaire has been used in the SOC PLUS model since its inception (2011): *Do you eat protein with every meal? Do you eat at least three times daily? Do you eat 3–5 servings of vegetables daily? Do you eat 1–2 servings of fruit daily? Do you drink sugar-sweetened beverages daily or weekly?* To ensure clarity, definitions of terms like protein, serving, and sugar-sweetened beverage were provided by a BCHN through nutrition education.
